# Supplementary figures and images for: Atypical cell cycle regulation promotes mammary stem cell expansion during mammary development and tumourigenesis
Source: Breast Cancer Res. 2024 Jun 28;26:106. doi: 10.1186/s13058-024-01862-1 (PMC11212383; doi:10.1186/s13058-024-01862-1)

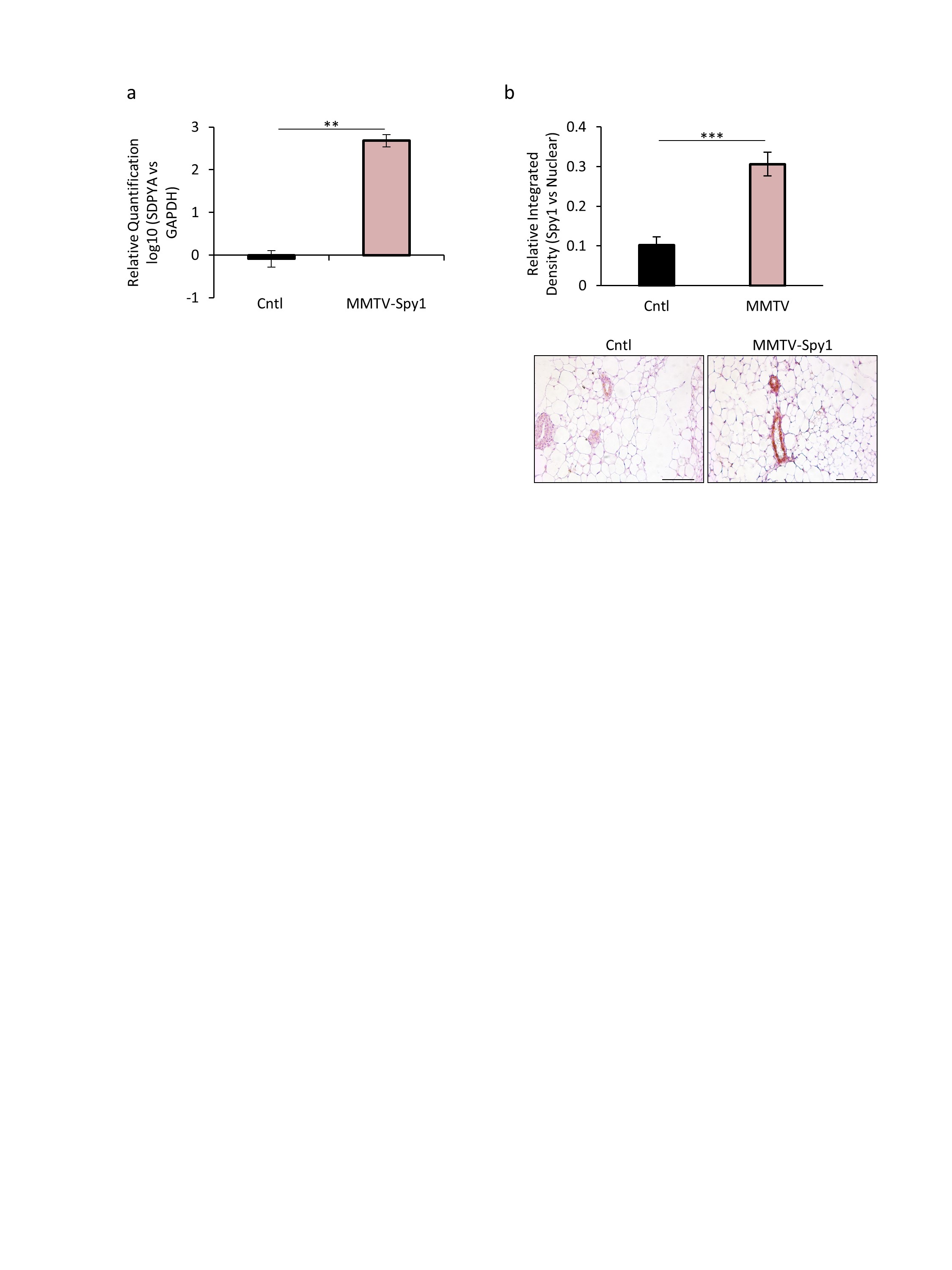

Supplement: Supplementary file 1 — Supplementary Figure 1: Spy1 is overexpressed in MMTV-Spy1 mammary glands. (a) qRT-PCR analysis of inguinal mammary glands from MMTV-Spy1 and littermate controls (Cntl) for Spy1 levels corrected for GAPDH (n = 4). (b) Spy1 expression in inguinal mammary glands collected from 8-week-old MMTV-Spy1 and littermate control (Cntl) mice. Representative images in bottom panels with quantification of Spy1 levels using ImageJ software analysis shown in the top panel. Scale bar = 100 µm (n = 3). Errors bars represent SE; Student’s T-test. *p < 0.05, **p < 0.01, ***p < 0.001 [file 13058_2024_1862_MOESM1_ESM.jpg]

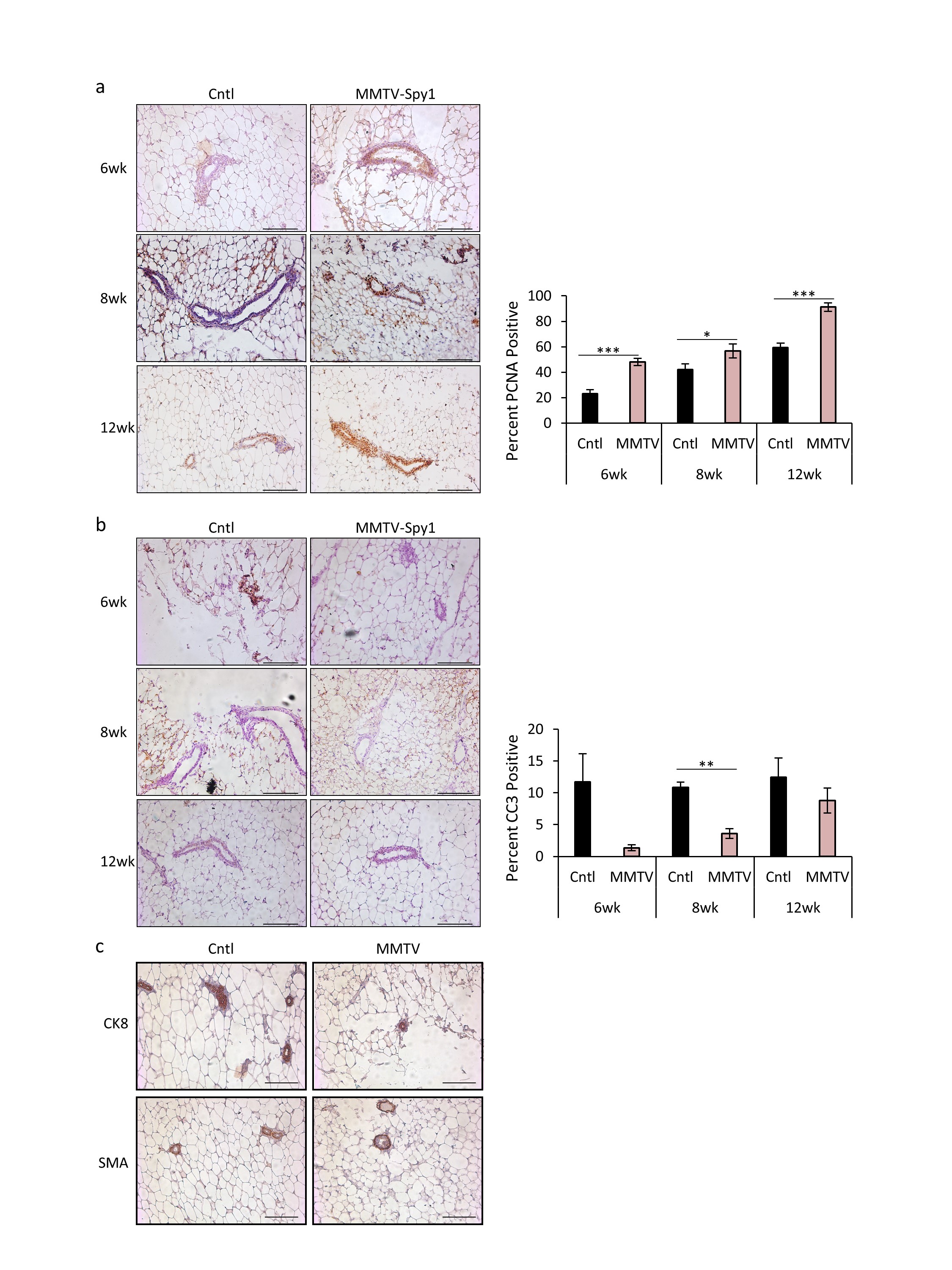

Supplement: Supplementary file 2 — Supplementary Figure 2: Spy1 increases proliferation and decreases apoptosis. (a) PCNA expression in MMTV-Spy1 and littermate controls via immunohistochemical analysis. Quantification of percentage of PCNA positive mammary epithelial cells over five fields of view per sample. Scale bar = 100µM (b) Cleaved caspase-3 (CC3) expression in MMTV-Spy1 and littermate controls via immunohistochemical analysis. Quantification of percentage of CC3-positive mammary epithelial cells over five fields of view per sample. Scale bar = 100µM. (c) Representative images of immunohistochemical analysis of CK8 and SMA. Scale bar = 100µM. n = 3; Error bars represent SE; Student’s T test. *p < 0.05, **p < 0.01, ***p < 0.001 [file 13058_2024_1862_MOESM2_ESM.jpg]

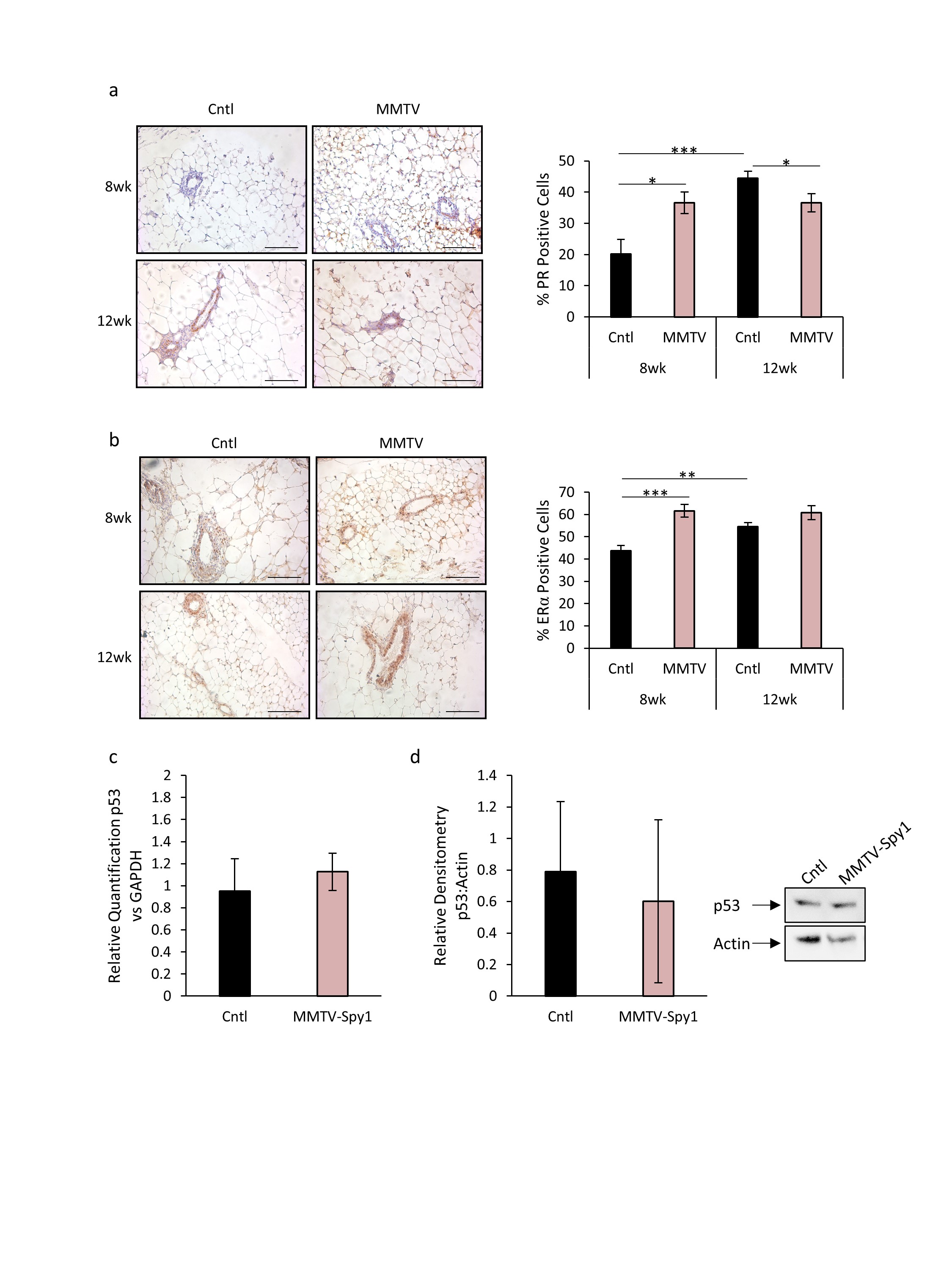

Supplement: Supplementary file 3 — Supplementary Figure 3: Altered hormone receptor status with elevated Spy1. Immunohistochemical analysis of (a) PR and (b) ER in 8- and 12-week-old MMTV-Spy1 and littermate control inguinal mammary glands. Representative images shown in left panels, where blue represents hematoxylin nuclear stain and brown represents (a) PR or (b) ER. Quantification of the percent positive (a) PR and (b) ER cells are depicted in right panels. Scale bar = 100 µm; (c) qRT-PCR analysis of inguinal mammary glands from control (cntl) and MMTV-Spy1 mice of p53 levels corrected for GAPDH. (d) Levels of p53 were assessed via western blot analysis in inguinal mammary glands of control (cntl) and MMTV-Spy1 mice. Left panel depicts densitometry analysis of p53 expression corrected for actin and right panel depicts representative blot. n = 3; Errors bars represent SE; Student’s T-test. *p < 0.05, **p < 0.01, ***p < 0.001 [file 13058_2024_1862_MOESM3_ESM.jpg]

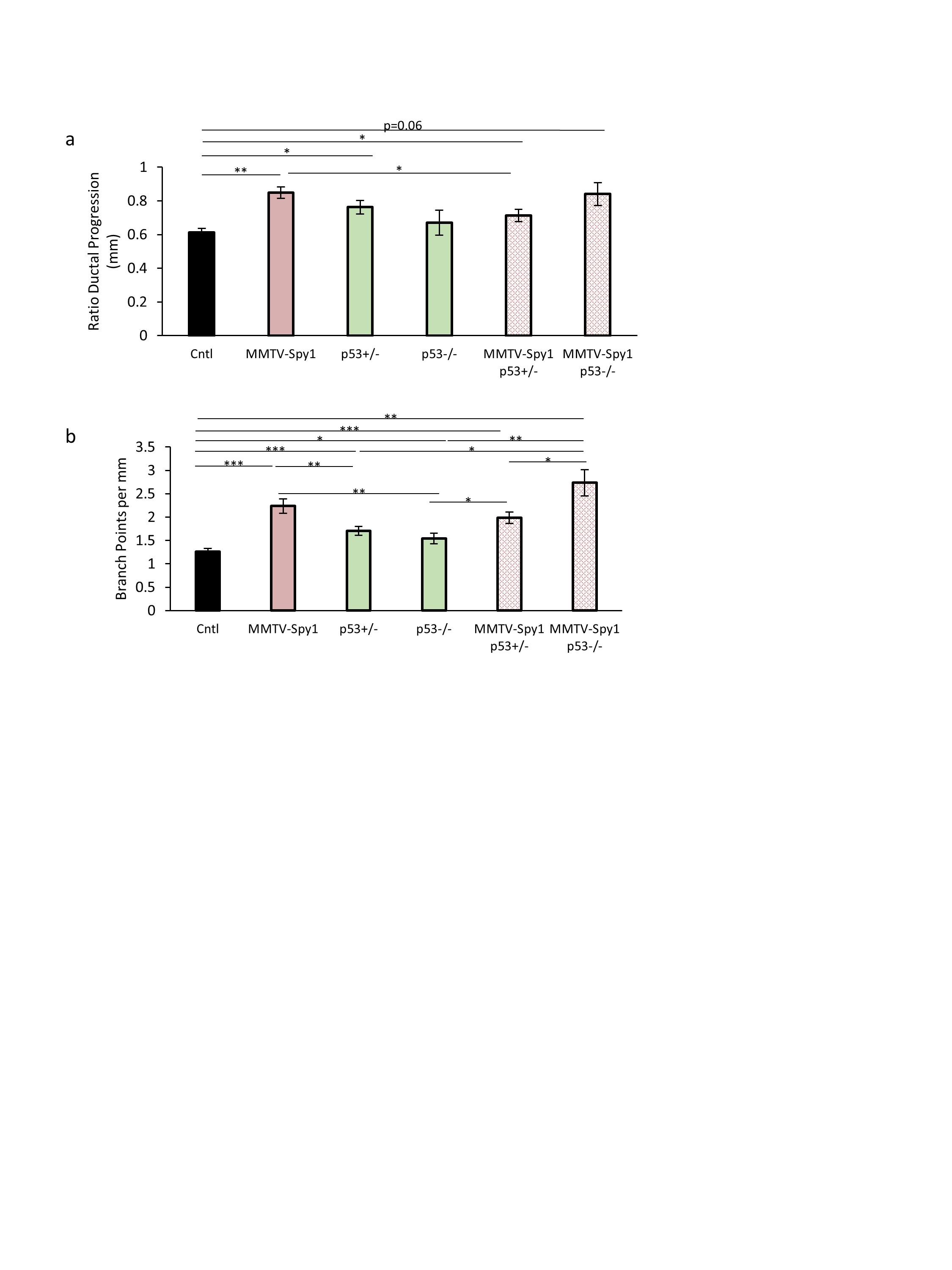

Supplement: Supplementary file 4 — Supplementary Figure 4: Loss of p53 does not alter Spy1 driven mammary development effects. Whole mount analysis was performed on inguinal mammary glands from 8 week old MMTV-Spy1 and p53 null intercrossed mice. (a) Ductal progression past lymph node and (b) number of branch points per mm of primary duct was quantified (Cntl n = 4, MMTV-Spy1 n = 5, p53+/- n = 5, p53-/- n = 4, MMTV-Spy1 p53+/- n = 7, MMTV-Spy1 p53-/- n = 3). Scale bar = 100µM. n = 3; Error bars represent SE; Student’s T test. *p < 0.05, **p < 0.01, ***p < 0.001 [file 13058_2024_1862_MOESM4_ESM.jpg]

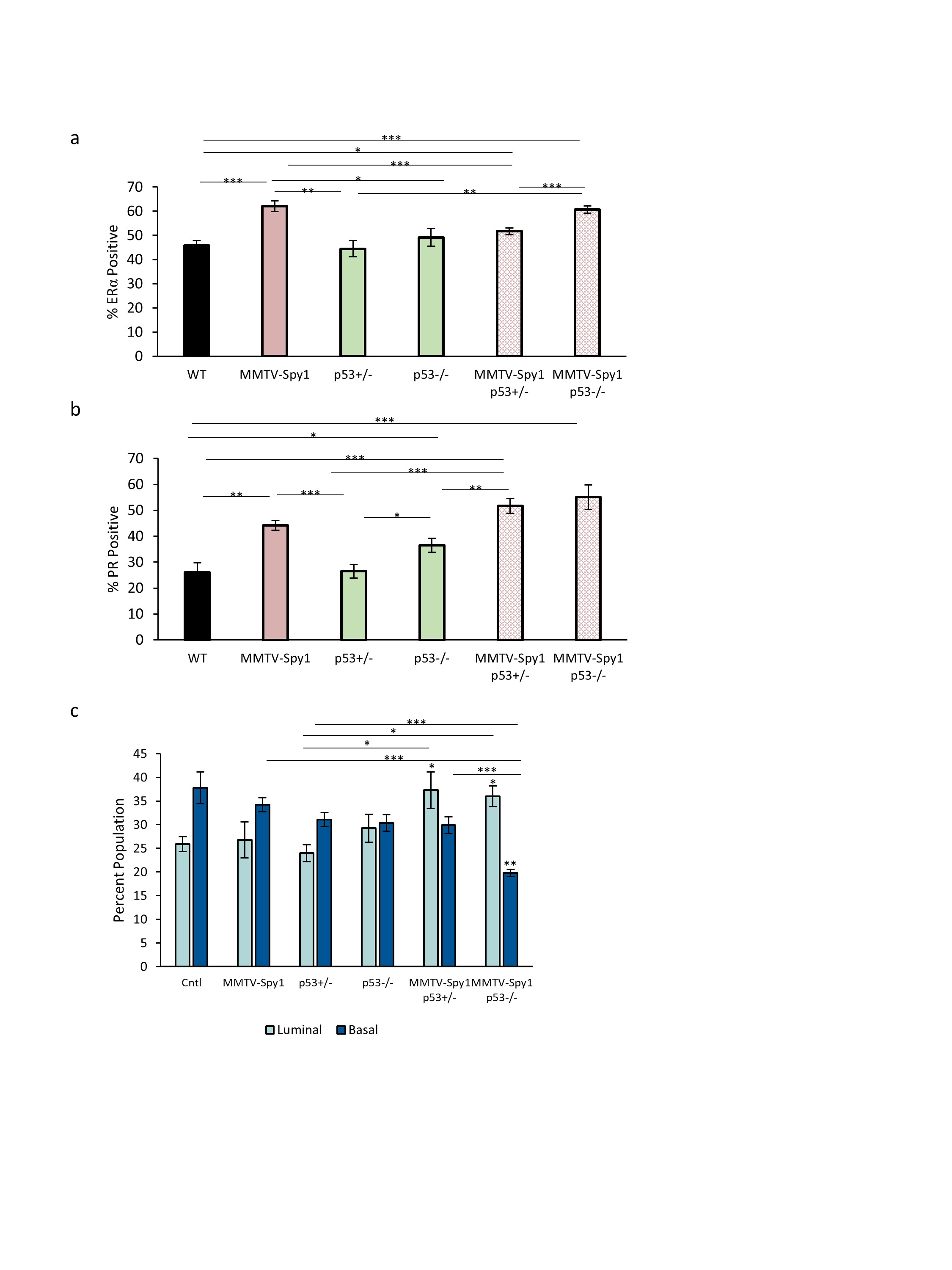

Supplement: Supplementary file 5 — Supplementary Figure 5: Loss of p53 does not alter hormone receptor expression in MMTV-Spy1 mice. Immunohistochemical analysis of the percent positive (a) ER and (b) PR in 8-week-old inguinal mammary glands of MMTV-Spy1 and p53 null intercrossed mice. Percent positive cells is represented graphically (Cntl n = 6, MMTV-Spy1 n = 7, p53+/- n = 4, p53-/- n = 3, MMTV-Spy1 p53+/- n = 6, MMTV-Spy1 p53-/- n = 3). (c) Flow cytometry analysis of primary mammary epithelial cells from inguinal glands of MMTV-Spy1 and p53 null 8-week-old intercrossed mice (Cntl n = 6, MMTV-Spy1 n = 8, p53+/- n = 6, p53-/- n = 3, MMTV-Spy1 p53+/- n = 8, MMTV-Spy1 p53-/- n = 4); Errors bars represent SE; Student’s T-test. *p < 0.05, **p < 0.01, ***p < 0.001 [file 13058_2024_1862_MOESM5_ESM.jpg]

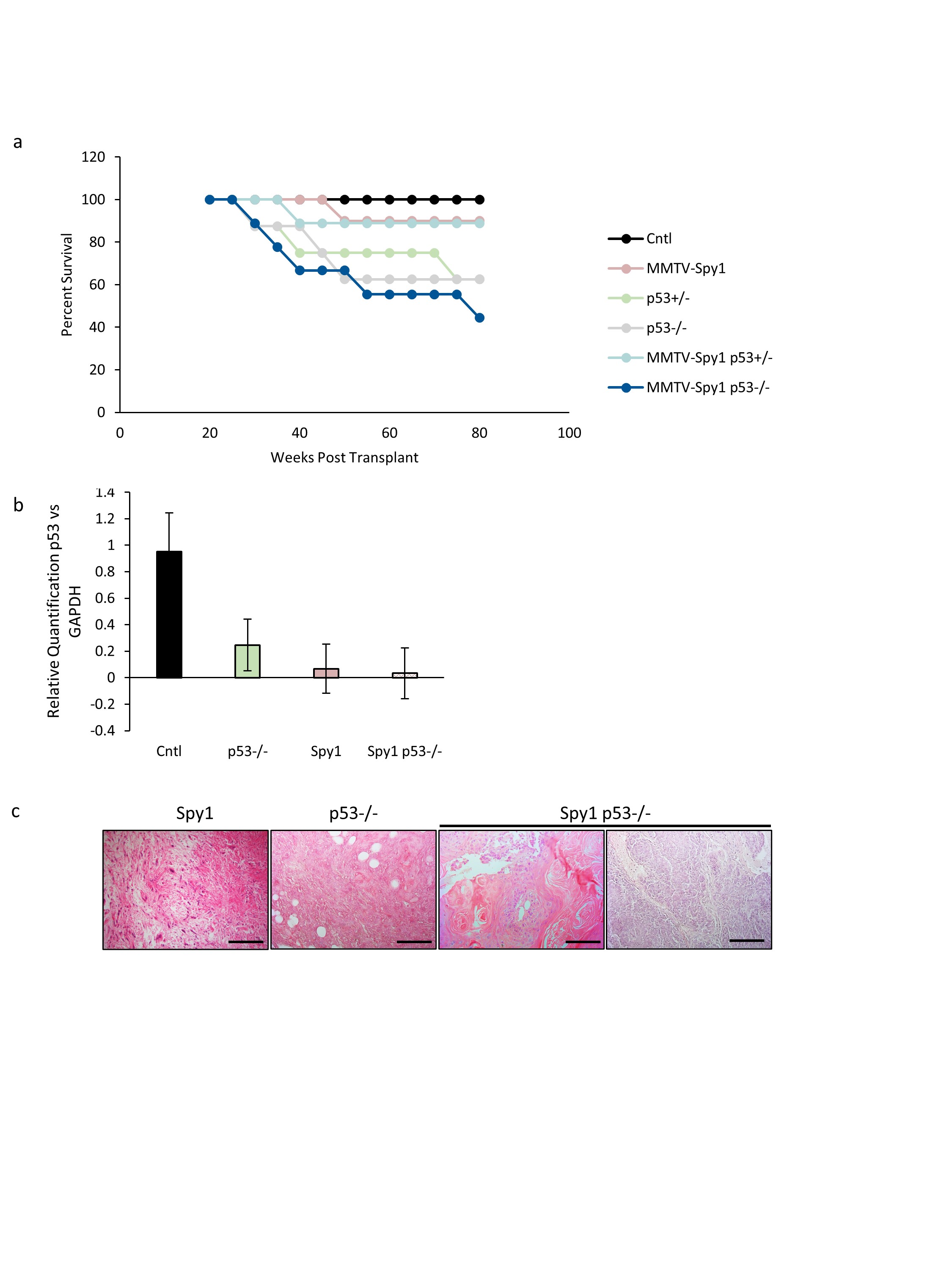

Supplement: Supplementary file 6 — Supplementary Figure 6: Primary mammary epithelial cells from MMTV-Spy1 and p53 null intercrossed mice were injected into the cleared fat pads of wildtype mice and monitored weekly for HANs and tumour development. (a) Timing of tumour onset is depicted. (b) qRT-PCR analysis of p53 levels corrected for GAPDH in Spy1, p53 null and Spy1 p53 null tumours (Spy1 n = 1; p53 null n = 3; Spy1 p53 null n = 3). (c) Representative hematoxylin and eosin stained images from Spy1, p53 null and Spy1 p53 null tumours. Scale bar = 100µM. (Cntl n = 15, MMTV-Spy1 n = 17, p53+/- n = 21, p53-/- n = 14, MMTV-Spy1 p53+/- n = 21, MMTV-Spy1 p53-/- n = 13) [file 13058_2024_1862_MOESM6_ESM.jpg]

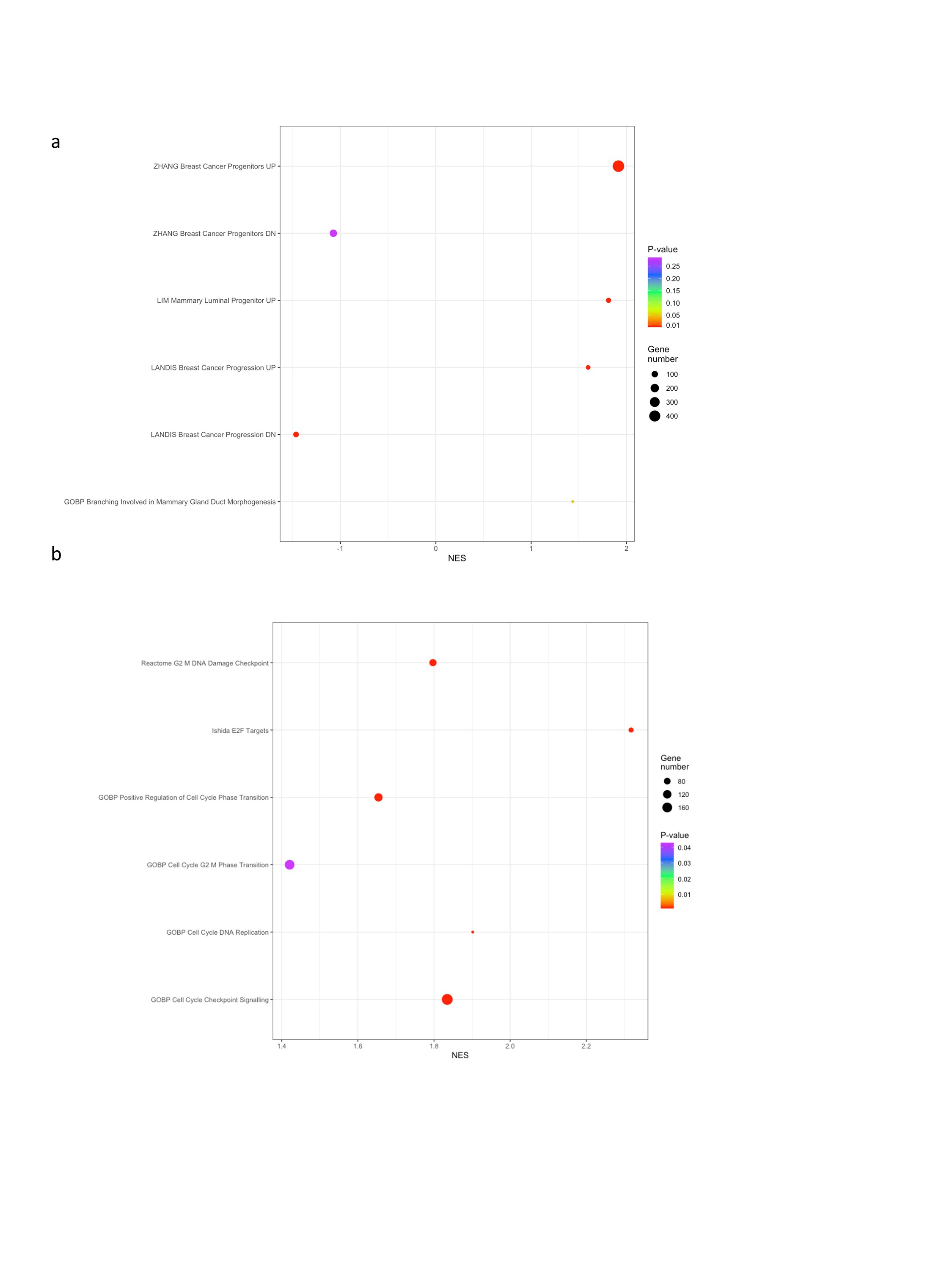

Supplement: Supplementary file 7 — Supplementary Figure 7: Dot plots depicting 2 sets of gene sets altered in Spy1 p53 null tumours as compared to p53 null tumours. Plots depict altered (a) breast specific gene sets and (b) cell cycle progression gene sets [file 13058_2024_1862_MOESM7_ESM.jpg]

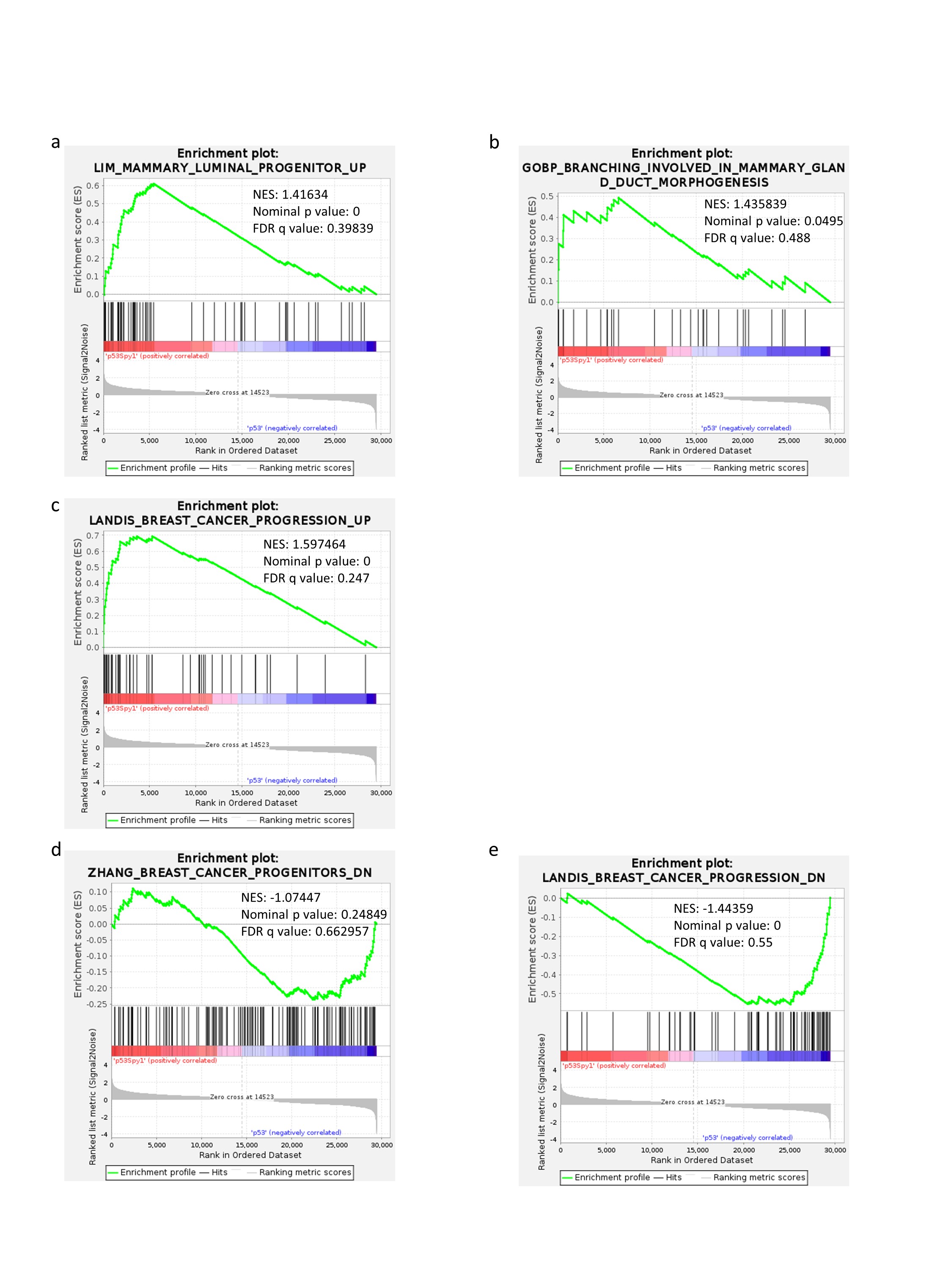

Supplement: Supplementary file 8 — Supplementary Figure 8: Spy1 driven tumours have enrichment in progenitor and breast cancer progression signaling. GSEA demonstrated that Spy1 p53 null tumours are enriched in (a) mammary luminal progenitors, (b) branching morphogenesis, and (c) breast cancer progression gene sets and are depleted for genes downregulated in (d) breast cancer progenitors and (e) breast cancer progression [file 13058_2024_1862_MOESM8_ESM.jpg]

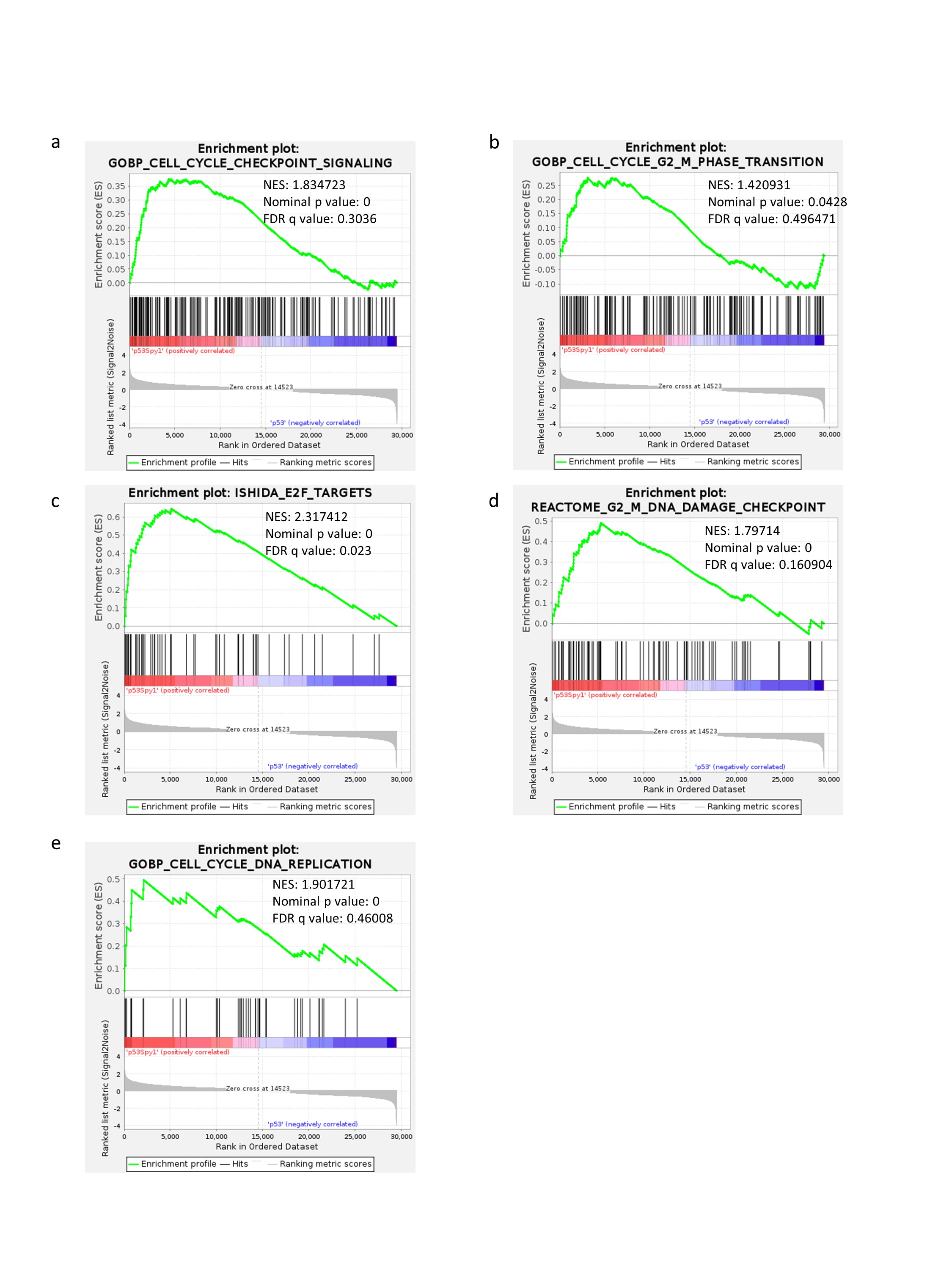

Supplement: Supplementary file 9 — Supplementary Figure 9: Spy1 driven tumours are enriched in cell cycle progression gene sets. GSEA demonstrated that Spy1 p53 null driven tumours are enriched in (a) cell cycle checkpoint signaling, (b) cell cycle G2 M phase transition, (c) E2F targets, (d) G2 M DNA damage checkpoint, and (e) cell cycle DNA replication gene sets [file 13058_2024_1862_MOESM9_ESM.jpg]

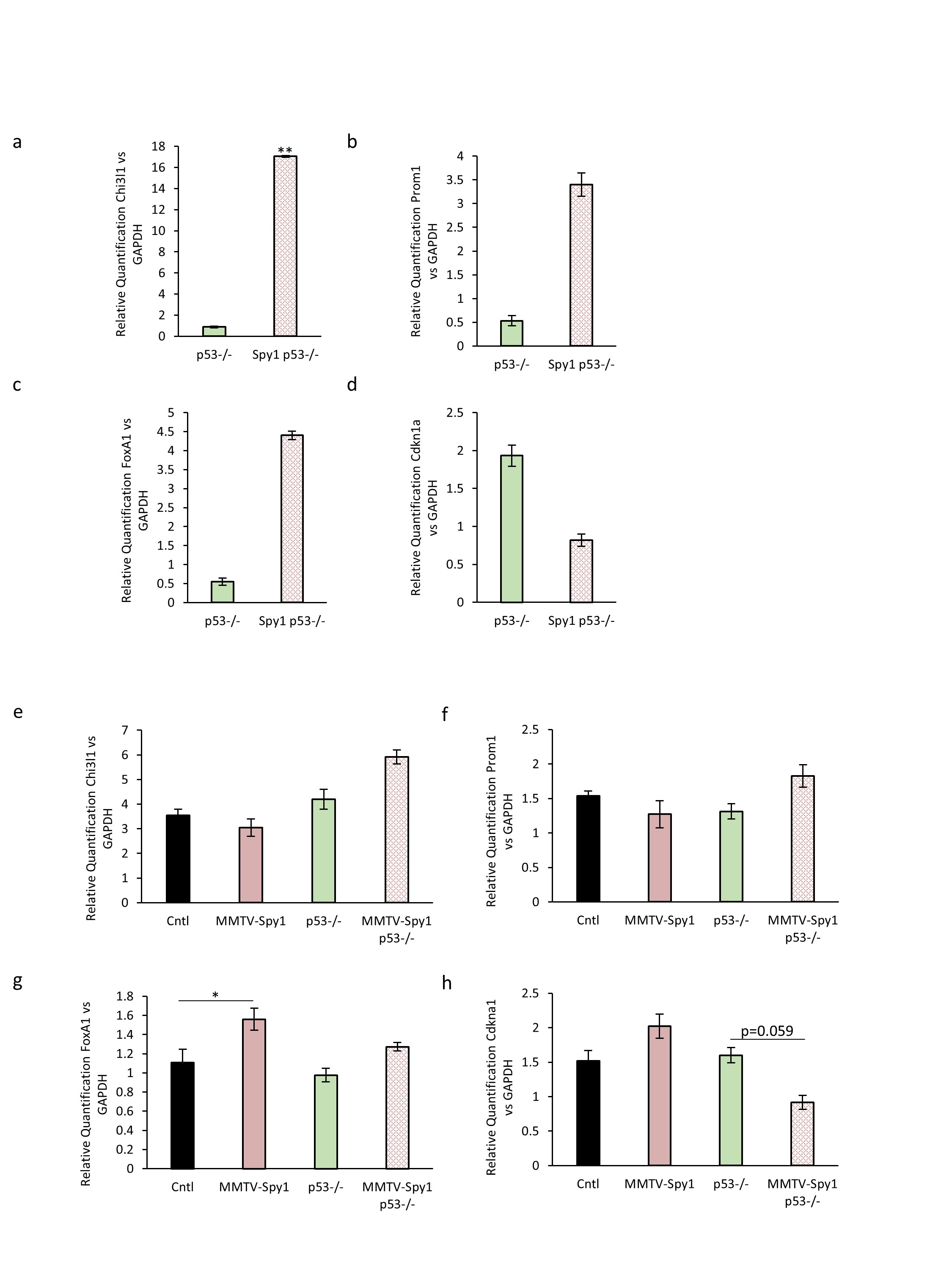

Supplement: Supplementary file 10 — Supplementary Figure 10: Changes in expression in Spy1 driven tumours are reflected during normal development. qRT-PCR analysis of expression of (a) Chi3l1, (b) Prom1, (c) FoxA1 and (d) Cdkn1a corrected for GAPDH in Spy1 p53 null and p53 null tumours (p53 null n = 3; Spy1 p53 null n = 5). qRT-PCR analysis of expression of (e) Chil3l1, (f) Prom1, (g) FoxA1 and (h) Cdkn1a corrected for GAPDH from inguinal mammary glands of 8 week old MMTV-Spy1 p53 null intercrossed mice (n = 3). Errors bars represent SE; Student’s T-test. *p < 0.05, **p < 0.01 [file 13058_2024_1862_MOESM10_ESM.jpg]

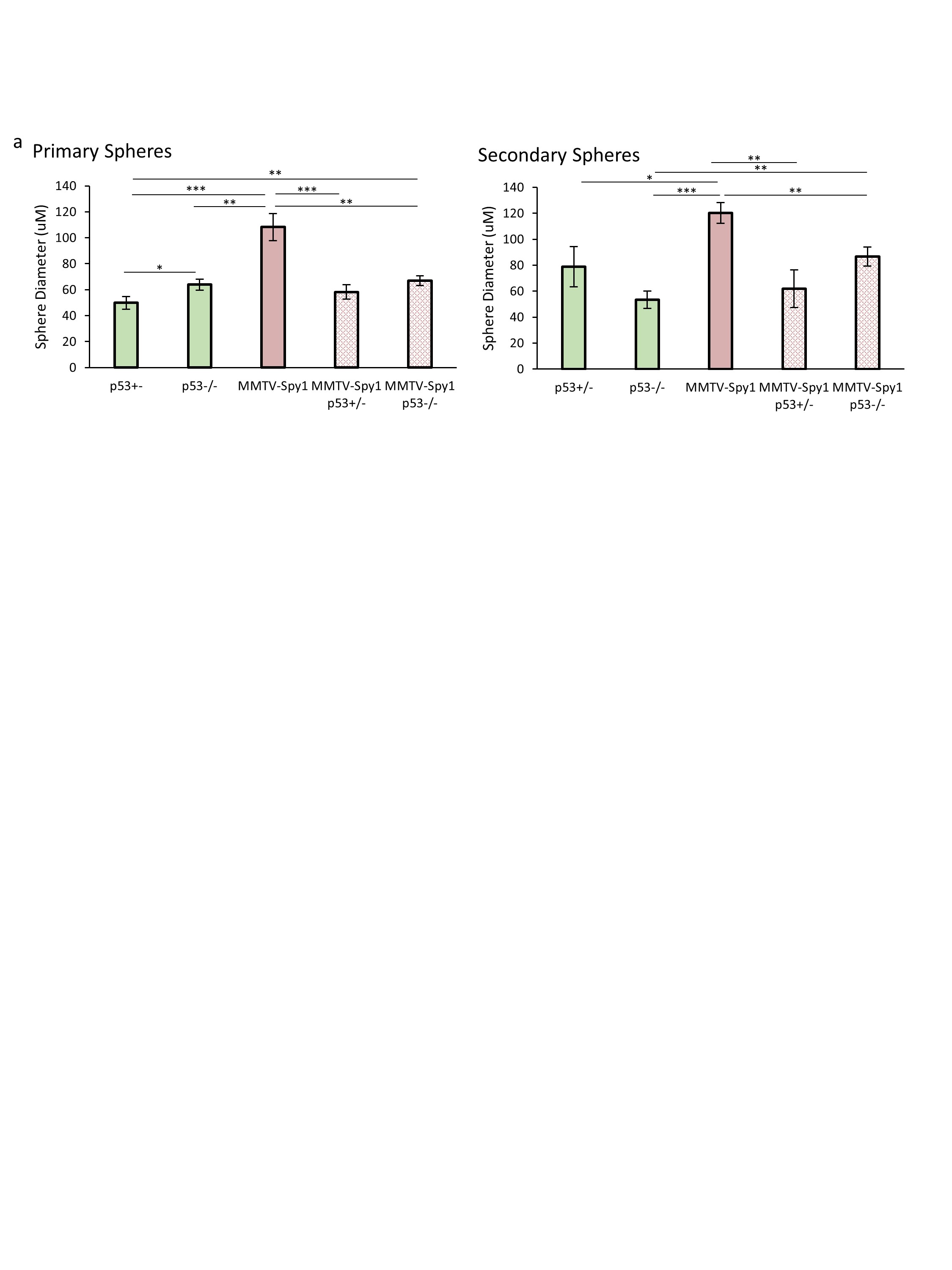

Supplement: Supplementary file 11 — Supplementary Figure 11: (a) Primary tumour cells from MMTV-Spy1 and p53 null tumours were cultured as mammospheres. Sphere diameter of primary (left panel) and secondary (right panel) mammospheres was quantified. N = 3; Error bars represent SE; Student’s T test. *p < 0.05, **p < 0.01, ***p < 0.001 [file 13058_2024_1862_MOESM11_ESM.jpg]

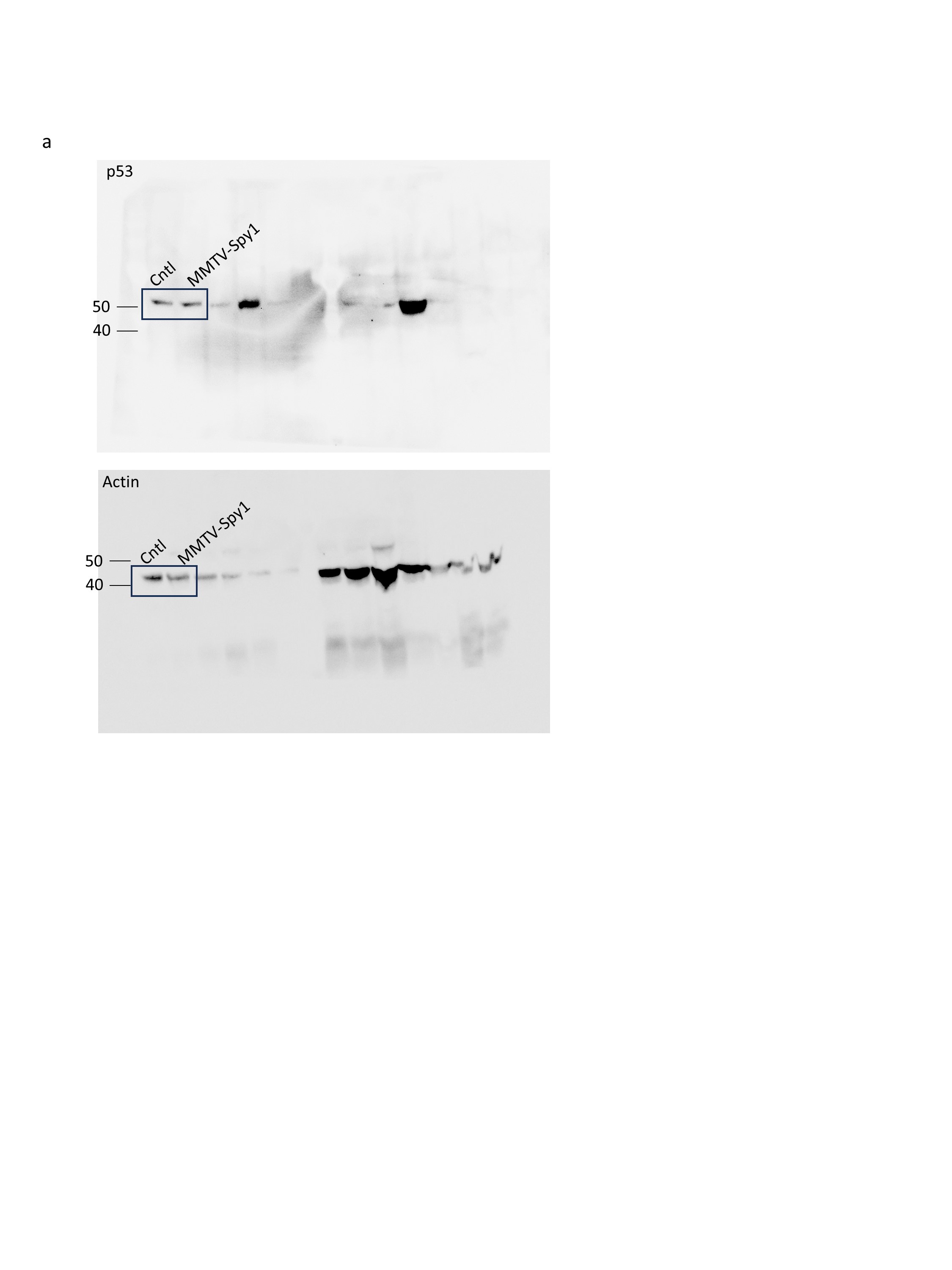

Supplement: Supplementary file 12 — Supplementary Figure 12: (a) Uncropped gel images from western blot images depicted in figure S3d [file 13058_2024_1862_MOESM12_ESM.jpg]
